# Some Methods of Separating Teeth with Wedges

**Published:** 1885-10

**Authors:** Dwight M. Clapp

**Affiliations:** Boston.


ARTICLE VII.

### SOME METHODS OF SEPARATING TEETH WITH WEDGES.

BY DR. DWIGHT M. CLAPP, OF BOSTON.

[Read at the joint meeting of the Massachusetts and Connecticut Valley Dental Societies, held at Worcester, Mass., June, 1885.]

Among the many disagreeable and annoying, not to say painful, things that patients have to suffer at the hands of dentists, nothing, perhaps, is received with greater dread and disgust than the announcement that the teeth must be "wedged" before filling. Some, a small minority among us, I think, always fill without previous separation. In regard to the necessity for it, I will enter no argument here, but only say that personally I am a firm believer in wide spaces between the teeth at their necks, and labor to the best of my ability to obtain this result. It is most likely that many of you are using the same means that I am to get the desired room for filling, but by presenting and discussing the subject, it is possible we may obtain some help in doing what I fear the most of us find, at times, difficult and perplexing. For a long time rubber was about the only thing used for separating. It has some good qualities and many bad ones. It probably causes more pain and annoyance to the patient than any other wedge. Its liability to slide into contact with the gum, causing great pain and soreness, and even suppuration, has caused me to entirely abandon its use. I am willing to admit that it may be used successfully sometimes. The best rubber to use, if it must be used at all, is that of which the most ine lastic tubing is made, or the erasers sold by stationers, cut into suitable shape. Wedges of wood are well adapted to cases where the sides of teeth to be wedged are nearly parallel, or where there is less space at the gum than at the points of the teeth. The wedge should be about as wide as the length of the crown, that is, it should extend from the cutting edge to the gum, nearly. It should be so shaped and trimmed as to not irritate the tongue or cheek. One advantage of the wooden wedge is that it is more cleanly than tape, cotton, or silk. This same class of teeth, those with nearly parallel sides, can be separated as successfully, and I think with less pain, with tape. Linen tape of various widths and well waxed is the best. It should be folded so as to be of proper width and thickness, and then drawn into place. A sharp knife is preferable to scissors for cutting off the ends. The tape should be thoroughly waxed, which assists materially in getting it between the teeth, and renders it more cleanly when left in the mouth for several days. In teeth with cavities so situated that cotton can be crowded in with sufficient force, this is one of the best wedges that can be used, as regards both effectiveness and comfort. It is necessary to so place the cotton that the force of expansion will be exerted against adjoining teeth and not expanded within the cavity. By once changing the cotton, space enough can generally be obtained. It is difficult to adjust and keep wedges in place between teeth having more or less space at the gum, and touching only at a small point near the cutting ends. It is in these cases that ligatures of various kinds serve an admirable purpose. Take for instance, the superior central incisors. These usually have but a small point of contact, with considerable space between them at the gum, and it is very difficult to put in a wedge of rubber, wood, or tape, that will not slip up against the gum, or come out altogether. If a ligature is used, the knots can be so tied that the string will clasp the point of contact in such a manner as to hold it quite firmly in place. There are many ways of making the knots; one is to pass the silk once between the teeth, then tie a surgeon's knot; but, before drawing it up, pass one of the ends again between the teeth, and then draw the knot so it will wedge from the gum towards the cutting ends; draw it closely, then finish by tying so that the last knot will be at the labial, or palatal side of the teeth. Another way is to make a series of knots like a chain stitch in crochet work, thus enlarging the silk for a suitable length; draw this between the teeth and tie as before, omitting the first knot that is drawn between the teeth. Another, and a very good way of enlarging the ligature, is, after well waxing it, to roll a little cotton around the silk as you would around a broach for wiping out a root canal, and draw this between the teeth and tie the same as when the silk is knotted. Still another method, easy of application and very effective in almost all cases where there is a cavity in one or both of the teeth, is to secure a pellet of cotton with the ligature. The silk is placed between the teeth in some of the before-mentioned ways; a pellet of cotton is forced into the cavity, projecting against the adjoining tooth, then the silk is tied firmly around the cotton. The swelling of the cotton and silk will make all the space necessary between any of the front teeth with but one application. The bulging of the cotton into the cavity or cavities, caused by tying the silk around it will hold it securely in place. This makes by far the most satisfactory wedge I have ever used, and, so far as I am aware, is original with me. It is sometimes well to open the cavity slightly with an excavator or chisel before wedging, so that the cotton will be more readily retained. For bicuspids and molars more than one application may be needed if much space is required. Quick wedging is sometimes possible, and when it can be done readily is usually desirable. Teeth that move easily may be separated sufficiently for operations by placing a wedge at the point of contact, and another near the gum, applying force gently with the hand, or light blows with a mallet, first on one, and then on the other, until wedged enough. Then remove the wedge that interferes most with the operation, leaving the other in place. Another way that often works well with children and with teeth that move readily, is to insert a large piece of rubber and let it remain from fifteen to twenty minutes, when the rubber will have opened a considerable space. A wooden wedge will keep the teeth from springing together while the work is being done. The appliances designed by Drs. Perry, Bogue and others, for making rapid separations, I have not used, but hear favorable reports in regard to them. Having spoken of rapid and semi-rapid separations, it is left only to speak of a methed which works very slowly. It applies, as a rule, to the biscuspids and molars only. In many cases where there are large cavities between these teeth, and often, when it is desirable that they should be filled with what I think is very properly called a "treatment filling," it is well to fill the entire space between the teeth with gutta-percha. In the course of a few months the process of mastication will force the gutta-percha toward the gum, and on removing what has not worn away the teeth will be found well separated, the cervical margins well in view, and the cavities in good condition for a metal filling.—*Archives of Dentistry.*

